# Adaptation and Outbreak of Highly Pathogenic Avian Influenza in Dairy Cattle: An Emerging Threat to Humans, Pets, and Peridomestic Animals

**DOI:** 10.3390/pathogens14090846

**Published:** 2025-08-25

**Authors:** Rifat Noor Shanta, Mahfuza Akther, M. Asaduzzaman Prodhan, Syeda Hasina Akter, Henry Annandale, Subir Sarker, Sam Abraham, Jasim Muhammad Uddin

**Affiliations:** 1 Department of Applied Animal Science and Welfare, Swedish University of Agricultural Sciences (SLU), SE-75007 Uppsala, Sweden; drrifat91@gmail.com; 2 School of Veterinary Medicine, Murdoch University, Perth, WA 6150, Australiaasad.prodhan@murdoch.edu.au (M.A.P.); hasina.akter@murdoch.edu.au (S.H.A.);; 3 Biomedical Sciences & Molecular Biology, College of Medicine and Dentistry, James Cook University, Townsville, QLD 4811, Australia; subir.sarker@jcu.edu.au; 4 Centre for Biosecurity and One Health, Harry Butler Institute, Murdoch University, Perth, WA 6150, Australia

**Keywords:** zoonosis, dairy cattle, mammary gland, cell tropism, milk production losses, One Health

## Abstract

Over the decades, cattle have not been considered primary hosts for influenza A viruses (IAV), and their role in influenza epidemiology has been largely unrecognized. While bovines are known reservoirs for influenza D virus, the recent emergence of highly pathogenic avian influenza (HPAI) H5N1 clade 2.3.4.4b in U.S. dairy cattle marks an alarming shift in influenza ecology. Since March 2024, this virus has affected thousands of dairy cows, causing clinical signs such as fever, reduced feed intake, drastic declines in milk production, and abnormal milk appearance. Evidence suggests that the virus may be replicated within mammary tissue, raising urgent concerns about milk safety, foodborne transmission, and occupational exposure. This review highlights the unprecedented expansion of viruses into bovine populations, exploring the potential for host adaptation, and interconnected roles of pets, peridomestic animals, and human exposure within shared environments. The potential impacts on dairy production, food safety, and zoonotic spillover highlight the urgent need for integrated One Health surveillance to stay ahead of this evolving threat.

## 1. Introduction

Avian influenza viruses, members of the *Orthomyxoviridae* family, are classified into four types—A, B, C, and D—based on the antigenic properties of their nucleoproteins (NP) [1,2,3]. Among them, influenza A viruses (IAVs) are the most concerning due to their broad host range and capacity for continuous genetic evolution, which allows them to cross species barriers and occasionally trigger pandemics [4,5]. Notable historical pandemics such as the 1918 H1N1 “Spanish flu” and the 2009 swine-origin H1N1 involved avian or swine intermediaries before adapting to humans. In contrast, influenza B viruses are mostly restricted to humans, while influenza C and D affect children and cattle, respectively, with milder pathogenicity [6,7]. The recent outbreaks have focused on the highly pathogenic avian influenza (HPAI) A/H5N1 clade 2.3.4.4b, one of the most virulent lineages known to affect birds and mammals [8]. First identified in poultry in East and Southeast Asia around 2020, this clade has since then spread globally via wild birds and trade networks [9]. In 2021, it reached North America, causing widespread outbreaks in wild birds and spillovers into terrestrial mammals, raising alarms over its zoonotic potential [10,11].

A notable shift occurred in March 2024 when HPAI H5N1 was detected in dairy cattle in Texas, marking the first such event in U.S. history [12]. By the end of May 2025, over 1072 dairy herds in 17 states had been affected. The affected cattle exhibited a sudden decline in milk production, along with mild respiratory signs. This unusual clinical presentation has prompted renewed interest in the virus’s ability to replicate in mammary tissue. Interestingly, this possibility was postulated decades ago when the influenza A virus was detected in the mammary glands of cows and goats [13,14]. Furthermore, the detection of cat fatalities and peridomestic animal deaths on infected farms further highlights the potential for environmental contamination and interspecies transmission [11]. Subsequently, several human cases were confirmed among dairy workers who had been working on the dairy farms, followed by additional infections in other wild mammals [15,16,17]. This highlighted not just a zoonotic spillover but a potential early-stage crossing of the species barrier.

Thus, this outbreak is not simply a continuation of previous avian influenza narratives; it reflects an evolutionary leap in viral ecology. The ability of H5N1 clade 2.3.4.4b to infect, replicate, and potentially adapt within bovine hosts, combined with interspecies jumps and early human cases, makes this strain a critical public health threat. It demands urgent, interdisciplinary action across veterinary, human, and environmental health disciplines. This review explores the emerging role of cattle in the H5N1 clade 2.3.4.4b transmission cycle, highlighting a shift in influenza ecology. Beyond animal health, the implications for dairy production, food security, and zoonotic spillover are profound. We reviewed the virus’s host expansion, examined cross-species transmission pathways, and unraveled critical gaps in current surveillance systems. We highlight current surveillance blind spots and advocate for a coordinated, multidisciplinary One Health strategy to monitor viral evolution and prevent future public health crises.

## 2. Literature Search

Although this is a narrative review, we endeavored to follow the PRISMA guidelines as closely as possible. Relevant literature, reports, and news articles were searched and screened using the following databases: Google Scholar, SCOPUS, PubMed, ScienceDirect, and the Science Citation Index Expanded (SciSearch). Boolean keyword search terms included: “bovine flu”, “highly pathogenic avian influenza”, “HPAI in cattle”, “H5N1 in cows”, “flu in dairy cows”, “avian influenza in dairy cattle”, “avian influenza AND milk drop”, “clade 2.3.4.4b in cattle”, “D 1.1 in dairy cattle”, “AIV in milk”, “bovine flu in cats”, “avian influenza spillover”, “H5N1 in dairy workers”, “avian influenza in cattle AND human”, “avian influenza in dairy products”, “avian influenza AND udder”, “flu virus in mammary gland”, “H5N1 in domestic AND peridomestic animals”, “flu vaccine in cattle”, and “avian influenza diagnosis”. It is worth noting that several challenges were encountered. For example, the unprecedented outbreak of influenza in bovine species in the USA is a recent event, meaning that limited research articles were available during the drafting of this review (January–May 2025). Therefore, in addition to research articles, we included review articles and reports from various government and non-government agencies, including state veterinary services, the CDC (Centers for Disease Control and Prevention), the WOAH (World Organization for Animal Health, formerly OIE), academic and research institutes, and reputable news outlets. Excluding news sources and relying solely on research articles would have resulted in missing valuable and relevant information.

While numerous scientific articles exist on avian and swine influenza due to their decades-long circulation, our focus was specifically on avian influenza in bovine species. Consequently, all original research articles, review articles, reports, and news items relevant to the scope of this review were included. Articles or reports published in non-English languages were excluded. Duplicate references were removed using the Mendeley reference manager, and discrepancies were resolved through cross-screening and collective discussion. In addition to highlighting the impact of avian influenza on the dairy industry, we also provide a brief description of the virus structure. Notably, the recent bovine flu outbreak has so far been reported only in dairy cows, peridomestic animals, and pets associated with dairy farms in various states of the USA, with the exception of a few sporadic historical cases. Nonetheless, many livestock-producing countries remain on alert and continue to monitor emerging information closely.

## 3. Influenza A Virus: Structure, Ecology, and the Threat of Host Expansion

**Figure 1:**
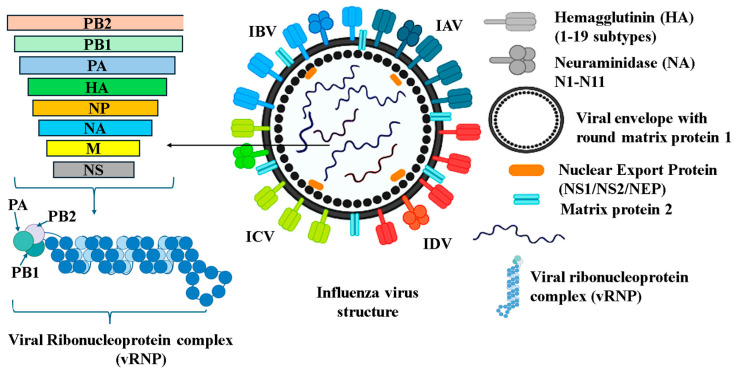
Schematic structure of influenza A virus (IAV). The viral envelope is derived from the host cell plasma membrane, which includes three transmembrane proteins: two surface glycoproteins, referred to as hemagglutinin (HA) and neuraminidase (NA), and the proton channel matrix protein 2. The matrix protein lies beneath the inner surface of the viral envelope and correlates with the NEP (Nuclear Export Protein) and viral ribonucleoprotein complex (vRNPs). The eight vRNPs comprise eight negative-strand RNA segments with nucleoprotein (NP) and three RdRp polymerase subunits (PA, PB1, PB2).

Influenza A viruses (IAVs) possess a segmented RNA genome, which allows them to exchange genetic material when two or more strains infect the same host. This reassortment process is a major driver of viral evolution and has been central to the emergence of past pandemics [18,19,20]. IAVs are classified into 19 hemagglutinin (HA) and 11 neuraminidase (NA) subtypes based on surface proteins (Figure 1). While most subtypes circulate in various hosts, H17N10 and H18N11 are unique to wild aquatic birds, which serve as their natural reservoir [21,22,23]. The IAV’s segmented genome not only enhances its adaptability but also poses a constant risk of generating novel, potentially pandemic strains [4]. Migratory waterfowl and shorebirds are key global reservoirs, with avian-origin strains giving rise to most mammalian influenza outbreaks [24]. IAVs predominantly persist in wild birds, often transmitted via fecal contamination in shared habitats along migratory routes [25]. Viral entry begins when hemagglutinin (HA) binds to sialic acid (SA) receptors on epithelial cells. Species-specific infection is influenced by SA types and their glycosidic linkages: human-adapted IAVs prefer α-2,6-linked SA, while avian strains bind α-2,3-linked SA [4,26,27,28].

**Figure 2:**
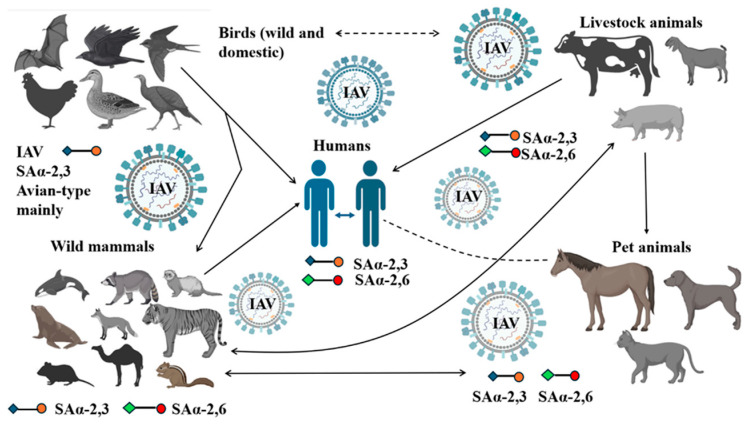
Interspecies transmission and host range of influenza A virus. Wild aquatic birds are the reservoir hosts for type A avian influenza viruses. Wild birds, wild mammals, livestock animals, and domestic/pet animals, as well as humans, have been affected during the recent (2024–2025) spillover of HPAI H5N1 clade 2.3.4.4b. Both α-2,3 and α-2,6 receptors were found in cattle and pigs.

Alarmingly, recent HPAI H5N1 outbreaks in U.S. dairy cattle reveal the virus’s ability to adapt in bovine hosts. High levels of α-2,3 and α-2,6 receptors suggest expanding the host range [29]. This dual receptor binding capability raises concerns about potential transmission to humans, underlining the urgent need for heightened surveillance across livestock interfaces. One of the most alarming features of IAV is its ability to adapt and jump between species (Figure 2). The classic example is the H1N1 strain, which originally came from the 1918 human pandemic and has since continued circulating in pigs [4]. In 2009, a new pandemic strain (H1N1/2009) emerged from pigs, formed through genetic mixing of several influenza lineages, including European avian-like swine, North American triple reassortment H1N2, and Eurasian avian strains, making it highly adaptable to humans [30,31]. Historically, cattle were not considered important hosts for IAV, with only mild infections reported in the 1970s. However, recent findings of H5N1 in dairy cows, with the virus found in respiratory and mammary glands, mark a concerning shift. These bovine viruses show the ability to bind both avian- and human-type receptors, suggesting they could infect a broader range of species [32,33]. This raises the possibility that cows, long overlooked in influenza research, could become key players in future outbreaks.

## 4. Unprecedented Emergence of Influenza in Bovine Hosts

IAV is best known for its ability to cause human pandemics, but its neglected history in cattle reveals a broader, more complex story of cross-species transmission. Initial outbreaks, such as the 1949 influenza-like illness in Japan, affected over 160,000 cattle [34], and subsequent events in Hungary (1962) [35] and the former USSR (1969) [4] demonstrate exposure of cattle to H1N1 and H3N2 subtypes. Notably, the H3N2 strain in cattle closely resembled the human pandemic strain A/Hong Kong/1/1968, suggesting a rare historical case of reverse zoonosis, but has no link to any current outbreaks [36]. Experimental infections highlight differences in host response; A/Aichi/2/68 showed no clinical signs in calves, while A/calf/Duschambe/55/71 induced influenza-like illness and persisted longer [37]. Notably, antigenically similar human-derived H3N2 viruses were subsequently identified in Russia and Hong Kong [38,39].

After a period of limited research, bovine influenza re-emerged in the late 1990s in the UK, affecting milk yield and health [40,41], with 2008 surveys confirming elevated H1N1 and H3N2 antibodies in Holstein Friesian cows [42]. Historically, influenza-like illness in cattle was typically characterized by mild symptoms, often described anecdotally or detected only by serology, which lacked virus isolation or genetic confirmation [33]. This is attributed to the presence of the influenza A virus, uncertain in most cases. By contrast, the U.S. dairy outbreak beginning in March 2024 represents a well-documented, evolutionary novel spillover of genotype B3.13, followed by onward bovine-to-bovine spread across multiple states [43]. Genetic analysis confirmed a single wildlife-derived introduction of clade 2.3.4.4b with evidence of reassortment and mammalian-adaptive mutations in both cattle and associated human cases. Compounding the complexity, a second independent spillover of D 1.1 was detected in Nevada in early 2025, phylogenetically distinct from B3.13 and traced back to wild birds via milk-screening surveillance [44]. What was once an ambiguous pattern of mild bovine illness has now been traced to two genetically distinct, well-characterized H5N1 introductions into dairy cattle. This finding highlights the likelihood of influenza reservoirs emerging in this species and heightens the urgency for strengthened cross-species surveillance and biosecurity.

## 5. From Birds to Mammals: Cross-Species Spillover of HPAI H5N1 with Emphasis on U.S. Dairy Herds

**Figure 3:**
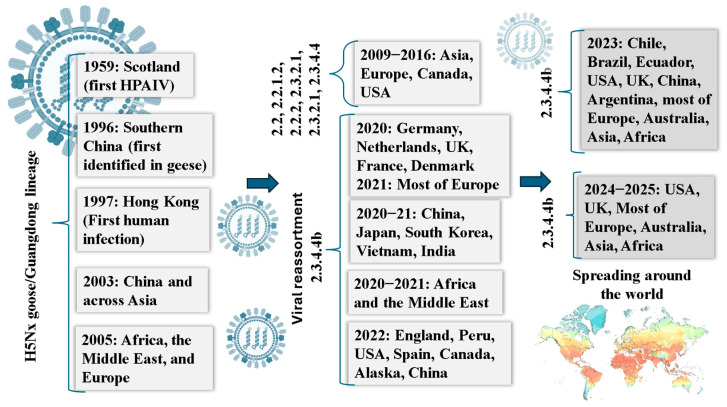
Timeline of Highly Pathogenic Avian Influenza (HPAI) H5N1 virus evolution (1959–2025). The timeline showcases significant events and outbreaks [48] associated with the virus, along with the corresponding countries and virus clades involved.

The ongoing global crisis caused by the HPAI H5N1 virus has its roots in the A/goose/Guangdong/1/96 (Gs/Gd) lineage, which has triggered the most devastating avian epidemic ever recorded in the wild bird population [45]. Although H5N1 was initially identified in poultry in Scotland in 1959, the outbreak in humans in Hong Kong in 1997 highlighted the devastating zoonotic potential [3,46]. Between 2003 and 2008, the virus escalated across Asia and beyond (Figure 3), becoming endemic in several countries, including China, Vietnam, Cambodia, Indonesia, Bangladesh, and Egypt [47]. Previously rare in wild birds, the virus underwent a genetic shift, gaining the ability to transmit in free-ranging avian populations. From 2021 to 2022, outbreaks were reported in 54 countries, and in the United States alone, over 800 poultry flocks and more than 7000 wild birds were infected across all 50 states between 2022 and 2023 [48]. The virus has jumped interspecies boundaries, posing a significant concern for spillovers into mammals, including foxes, seals, and farmed mink, which were documented in multiple regions, signaling a wider host range and raising concern over viral adaptation to new species. Alarmingly, 878 human HPAI H5N1 infections were confirmed globally between 2003 and mid-2023, with a staggering 52% case fatality rate [48]. As infection pressure intensifies within avian populations, the likelihood of adaptation to new hosts, including economically vital livestock and humans, increases dramatically [49]. The following subsections address both documented spillovers in mammalian hosts broadly and specific considerations for cattle, given their agricultural and public health relevance.

### 5.1. Transmission Pathways and Interspecies Spread of HPAI H5N1 Clade 2.3.4.4b in U.S. Dairy Cattle

The emergence of HPAI H5N1 in U.S. dairy cattle marks a paradigm shift in our understanding of influenza virus ecology, raising important questions about transmission dynamics, host adaptation, and cross-species spillover. The first confirmed case of H5N1, specifically clade 2.3.4.4b genotype B3.13, was reported in January 2024 in Texas, USA, with subsequent spread to over 17 states, including Kansas, Michigan, and Ohio, by early 2025 [50,51]. However, a recent epidemiological model study revealed that the actual dairy herd infections may be four times higher than official counts, estimating approximately 4000 infected herds compared to around 1000 reported. The model also finds that current intervention efforts have only prevented about 175 reported outbreaks, underlining the urgent need for more proactive farm-level surveillance and biosecurity measures [52]. A notable delay in clinical sampling and diagnostic testing during the early stages of the outbreak likely contributed to the virus’s silent establishment within vulnerable dairy herds, underscoring gaps in current surveillance systems.

The infected cows predominantly exhibited symptoms such as fever, respiratory distress, lethargy, nasal discharge, and a sharp decline in milk production [53,54]. One of the most characteristic clinical findings was abnormal milk, often yellowish, thickened, or curdled, linked to mammary gland pathology, including tissue involution [53]. Viral RNA and infectious particles were found in milk but were notably absent from nasal swabs or blood beyond 16 days post-clinical diagnosis [11]. This finding suggests a unique tropism of the H5N1 virus for the bovine mammary gland, distinguishing its transmission dynamics from traditional respiratory pathways observed in avian and human infections. Additionally, in a Michigan dairy herd, HPAI H5N1 infected 32% of cows over 45 days, resulting in a 7% decrease in milk yield and prolonged declines in dry matter intake and herd productivity [55].

**Figure 4:**
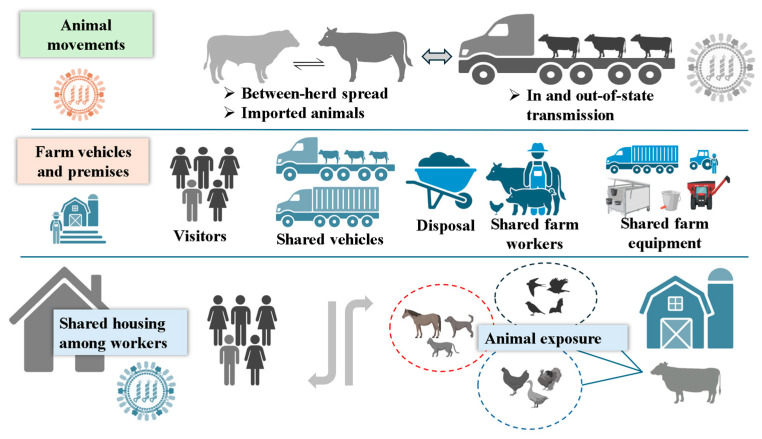
Possible transmission dynamics of HPAI H5N1 in dairy cattle and interspecies transmission. Transmission of viruses can occur in several ways, including animal movements, farm vehicles, contaminated farm environment, exposure from nearby farms or wild or open environments, and shared housing of animals or workers.

The initial event for the bovine spillover was suspected to have been infected wild birds, as concurrent avian mortality events were documented on the affected properties [53]. However, cow-to-cow transmission has become increasingly evident, likely mediated through contaminated milking equipment, shared water supplies, and close-contact housing facilities [21,56]. Notably, contamination of farm environments via feces and raw milk shedding of virus through fomites and indirect contact routes are thought to be the ways of virus spread within and potentially between herds. While airborne transmission cannot be entirely ruled out, the predominant route of cow-to-cow transmission appears to be iatrogenic, especially via milking apparatus and contaminated raw milk. Given the broad environmental persistence of IAVs, shared housing with other livestock, contact with wild birds, and movement of animals between farms remain critical epidemiological links in the transmission chain [57]. This risk is heightened in integrated farm systems where poultry and cattle are managed nearby, where shared equipment, uncleaned vehicles, and the movement of personnel between poultry and cattle operations could inadvertently facilitate the transfer of H5N1 (Figure 4).

Furthermore, the virus has demonstrated cross-species spillover capacity within affected farms. Cats fed with raw, unpasteurized milk from infected cows developed neurological signs and died in high numbers, with over 50% mortality reported in some herds [1]. Viral RNA was isolated from the brain and lung tissues of these cats, supporting systemic dissemination. Peridomestic species such as chickens, pigeons, raccoons, and foxes were also affected, highlighting the multispecies susceptibility and ecological complexity of this outbreak [54]. The confirmation of the H5N1 D1.1 genotype in dairy cattle in Nevada in January 2025 signified a second independent spillover event, likely from wild migratory birds [15]. This newly emerged genotype raises concerns about viral reassortment and evolution in mammalian hosts. Importantly, human infections have been reported among dairy workers, though clinical symptoms have remained mild (e.g., conjunctivitis) in most cases, contrasting with the severe respiratory outcomes documented in poultry workers infected with similar genotypes [15,58]. Nevertheless, the current H5N1 outbreak in U.S. dairy cattle underscores the unprecedented expansion of host range and transmission complexity of avian influenza viruses. The evolving dynamics highlight the need for stringent biosecurity measures, routine milk testing and sequencing, and One Health surveillance systems that integrate animal, environmental, and human health data. Additionally, understanding of HPAI transmission in bovines is not only vital for safeguarding global food systems but also for mitigating future zoonotic threats. Notably, this outbreak of AIV in dairy cattle, with spillover to dairy workers and companion animals, represents an unprecedented event not previously documented anywhere. To date, cases have been confined to dairy farms within the United States. Nevertheless, the profound consequences for the dairy sector have raised global concerns for livestock industries and public health.

### 5.2. Rethinking Tropism: The Mammary Gland Plays a Critical Role in Virus Evolution and Transmission

**Figure 5:**
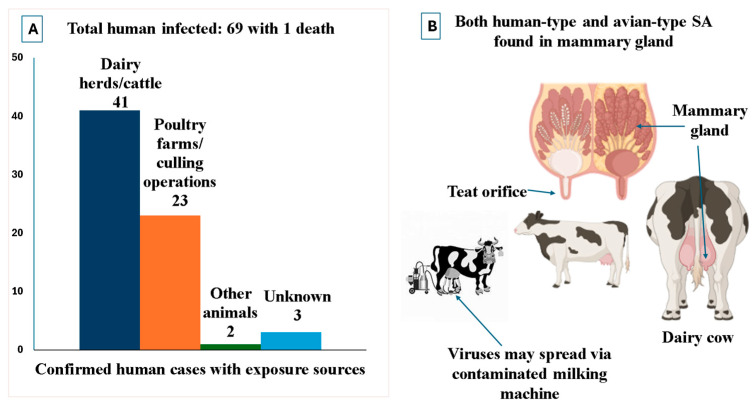
Confirmed human cases of HPAI H5N1 with exposure sources and mammary gland replication of the virus. (**A**) HPAIV H5N1 has been reported in humans, with the highest exposure from dairy herds compared to other sources. Dairy personnel who had been in close contact with infected dairy cows in the milking parlor or with contaminated dairy equipment without wearing any protective gear became infected. (**B**) Possible external source of causing HPAIV H5N1 virus entry and infection spreading in dairy cattle. The mammary gland is the most preferred location for virus replication and milk for viral shedding, which can enter through the teat orifice or teat canal during the use of the milking machine. Due to the pressure of the machine, the teat canal might not return to normal condition or might cause delayed closure of the teat orifice. This can facilitate the virus entering through the canal with other bacteria and cause infection.

The emergence of HPAI H5N1 clade 2.3.4.4b in North American dairy herds has prompted a rethinking of avian influenza viruses’ (AIVs) ecology, particularly regarding host range and tissue tropism. For decades, bovines were considered minor or accidental hosts for AIVs, with limited exposure to infection. Experimental intranasal inoculation of calves with H5N1, for instance, resulted in no viral shedding or clinical symptoms [59]. However, existing knowledge has changed dramatically due to the outbreaks in dairy cattle in the U.S., where viral RNA and infectious viruses have been recovered from the mammary gland, an unexpected site for AIVs [57,60]. Unlike the conventional respiratory focus of influenza surveillance, these infections have shown a remarkable mammary tropism, characterized clinically by mastitis-like symptoms, thick, yellow milk with clots, and confirmed virologically by high H5N1 loads in milk samples and mammary tissues. In situ hybridization and immunohistochemistry techniques have revealed viral RNA and nucleoprotein within the alveolar epithelial cells of the lactating mammary gland, as well as in the ducts and cistern epithelium [53,61]. The expression of both α2,3-linked (avian-type) and α2,6-linked (human-type) sialic acid receptors in bovine mammary epithelium offers a plausible explanation for this tropism and points to the gland as a site of potential viral adaptation across host species [61,62,63]. This biological vulnerability is magnified by dairy management practices, particularly mechanical milking (Figure 5). The repeated insertion of milking units and vacuum pressure may cause transient dilation or delayed closure of the teat canal, facilitating entry of viral particles into the gland [57]. Infected cows exhibit inflammation consistent with neutrophilic and lymphoplasmacytic mastitis, with histological evidence of cellular debris and alveolar damage [53]. These signs strongly indicate active replication and tissue destruction, rather than passive infection or collateral damage. The fact that HPAI H5N1 can infect the mammary gland shows it is no longer limited to birds or just the respiratory system. Instead, the virus is finding new places in the body to grow, which could change how it spreads and evolves. The mammary gland might even become a key site for the virus to multiply, transmit to other species, and develop new traits (Figure 5).

Importantly, the viral replication in bovines has real-world consequences beyond the herd. Human infections in dairy workers have been confirmed by the CDC, with viral genomic sequences closely matching those found in infected cows [64]. Most affected individuals were involved in direct animal handling or milking operations and did not wear appropriate personal protective equipment. The virus has also infected cats on farms, showing neuroinvasive disease and H5N1 presence in the brain, further confirming mammal-to-mammal transmission potential [53]. Recent ex vivo experiments using mammary gland and teat tissues from lactating cows demonstrated that H5N1 viruses originating from dairy cattle replicate far more efficiently in the glandular epithelium than avian- or human-origin influenza strains. This finding suggests the virus is adapting to mammalian hosts by exploiting the bovine udder as a specialized niche for replication [62]. Given the continued detection of viral RNA in raw milk and the potential for ingestion or aerosol exposure during milking, the risk of silent zoonotic transmission is now considered credible and significant [57,60]. The One Health implications are profound, particularly due to the recent emergence of genotype D 1.1 in dairy cattle from wild bird reservoirs, which may pose even higher risks to mammalian hosts [57].

### 5.3. Emerging Threat of HPAI H5N1 in Mammals with a Focus on Recent Feline Infections Linked to Dairy Cattle

HPAI H5N1 poses a great threat to the global poultry industry because frequent avian influenza outbreaks result in substantial economic losses for both the meat and egg-producing poultry industries. However, this review focuses on influenza infections in mammalian species, with a special interest in bovine flu. Nevertheless, HPAI H5N1 clade 2.3.4.4b has demonstrated the ability to infect a diverse range of wild and domestic mammals, including goats, sea lions, dolphins, foxes, bobcats, Virginia opossums, raccoons, coyotes, striped skunks, seals, black bears, polar bears, and tigers [65,66,67,68,69,70,71]. Notably, in 2004, two tigers and two lions in Thailand contracted the H5N1 virus after consuming raw, virus-contaminated chickens, providing strong evidence that ingestion of the virus-infected birds can facilitate virus transmission [72]. In 2022, HPAI H5N1 infections were confirmed in minks in Spain as well as in a dolphin and a sea lion in Peru, all of which exhibited respiratory and/or neurological symptoms leading to mortality [73]. Furthermore, in June 2023, multiple cats in Poland tested positive for the HPAI H5N1 virus, likely due to the consumption of contaminated cat food [74]. Similar infections in cats have also been reported in several countries, including Germany, the USA, and South Korea [21]. Additionally, a dog acquired the HPAI H5N1 virus infection in Canada in 2023 after chewing on a wild goose, indicating adaptability of this deadly H5N1 to mammals [75].

A particular concerning trend is the emergence of H5N1 infections in domestic cats, especially those associated with dairy cattle and raw pet food. Since 2024, over 200 H5N1 outbreaks have been reported, mostly from indoor cats on dairy farms in the U.S., indicating infection routes such as consuming unpasteurized milk, raw meat products, or indirect contact with contaminated farm environments [17,76,77]. Infected cats often show severe symptoms such as respiratory distress, neurological signs, ataxia, and rapid progression to death [78]. This evidence provides additional weight to the hypothesis that consumption of raw milk or uncooked meat is a significant route for H5N1, particularly clade 2.3.4.4b, transmission to cats and potentially other animals. However, a recent systematic review based on two decades of literature confirms that cats are not incidental hosts but are highly susceptible to severe, often fatal infections, including lung and brain, with H5 and H7 subtypes [79]. These infections can also be traced back to exposure to infected poultry or even raw milk from cows, suggesting a likely spillover from livestock to felines. Complementing these findings, another experimentally infected study demonstrated that H5N1 viruses isolated from cats in South Korea, carrying mammalian-adaptive mutations (PB2-E627K or D701N), exhibited fatal virulence in murine and ferret models, causing 100% mortality following intranasal inoculation [80]. Moreover, these viruses were capable of direct contact transmission among ferrets, confirming their high pathogenicity and mammal-to-mammal spread. Despite substantial evidence of feline susceptibility to AIV, spillover from cattle to cats has not been reported worldwide. Collectively, these data strongly support the notion that felines are susceptible hosts for HPAI H5N1, whereas cows and humans remain comparatively less affected.

While cases of cat-to-cat transmission have been documented [79], there is no evidence for cat-to-cow transmission, reinforcing the notion that the most likely direction of interspecies transmission in recent dairy farm outbreaks is from infected cattle to felines, particularly through contaminated milk, meat, or environmental exposure. Further strengthening this transmission model, several recent investigations have identified raw milk and meat as critical sources of infection. A recent CDC report from California confirmed that domestic cats developed fever and neurologic symptoms after ingesting raw milk contaminated with H5N1, with two cats dying and the surviving cat testing positive for H5N1 in urine [81]. In parallel, an experimental study showed that mice administered raw milk from infected dairy cows exhibited high viral loads in respiratory tissues, confirming that raw milk consumption can lead to systemic infection in a mammalian model [82]. Together, these findings underscore the fact that, while bovines and humans often experience subclinical or mild infections, cats and small mammals act as highly susceptible hosts and may serve as important amplifiers of the virus. This evolving dynamic emphasizes the need for strict control measures in dairy and poultry operations, along with heightened surveillance in domestic pets, to prevent further mammalian adaptation and cross-species spread.

Although there is no evidence of virus transmission from cats to humans, due to the evolving nature of the virus, these felines may pose not only veterinary challenges but also public health risks due to their proximity to humans. Additionally, the consistent appearance of severe neurological and respiratory signs with rapid death in cats suggests a particularly virulent phenotype of HPAI H5N1 in felines, which may be different from earlier clades. This could suggest adaptive evolution of the virus in mammalian hosts; however, further research is necessary. In addition, bovine H5N1 viruses have been isolated from various animals, including wild birds, terrestrial mammals, raccoons, and other carnivores living close to dairy herds. However, the exact mechanisms by which wild birds and other animals acquire these infections are largely unclear [83]. The identification of bovine H5N1 viruses in a diverse range of animals, including wild carnivores and domestic pets near dairy herds, suggests multi-host circulation in shared environments, indicating complex ecological spillover dynamics that remain poorly understood.

## 6. H5N1 in the Dairy Industry: Zoonotic Spillover and Impact on Public Health

The unprecedented emergence of H5N1 in the U.S. dairy production chain marks a pivotal shift in the ecology of zoonotic diseases, transforming milk into a possible carrier of viral threats. Unlike previous spillovers that were restricted to avian or swine reservoirs, this unexpected transmission exploits the lactational pathway with high concentrations of infectious virus through milk, ranging from 10^4^ to 10^8.8^ TCID_50_/mL in milk samples and up to 10^7.8^ TCID_50_/mL, indicating robust viral replication in mammary gland tissues within milk-secreting epithelial cells [84]. The viability of H5N1 viral particles in refrigerated raw milk for up to five weeks, and viral RNA persistence for eight weeks under cold chain conditions, suggests that milk may serve as a stable medium for prolonged infectivity beyond the initial contamination [33]. This thermal stability in raw milk raises urgent concerns for consumers, dairy producers, and foodborne disease surveillance systems. In experimental models, mice fed raw milk from HPAI H5N1-infected cows developed systemic infections, with viral replication confirmed in the respiratory, gastrointestinal, and neural tissues, demonstrating facilitated cross-species transmission in a novel ingestion pathway [85]. While commercial pasteurization processes, particularly High-Temperature Short-Time (HTST) treatments at 72 °C for 15–30 s, are generally effective in inactivating the virus, inconsistencies in time–temperature parameters, equipment precision, or the protective properties of fat globules in milk can lead to incomplete viral inactivation under certain conditions [33,85].

Notably, RNA fragments of the virus have been detected in approximately 20% of retail pasteurized milk samples in affected regions, suggesting the possibility of contamination during the pre-pasteurization process [33]. While attention has mostly been on raw milk as a medium of transmission, several investigations have focused on cheese and fermented dairy products as the underrecognized medium for viral persistence, especially when thermal processing was absent. The studies also revealed that certain cheeses, particularly soft, semi-soft, and fresh varieties made from unpasteurized milk, can support prolonged viral survival, challenging assumptions that aging, acidification, or fermentation alone are sufficient to kill the virus [86,87]. It is suggested that the virus benefits from high moisture content, acidity, and protective fat layers, which together create a microenvironment where viral particles can remain stable throughout the product’s shelf life. A recent U.S. surveillance study identified H5N1 genetic material in multiple aged raw-milk cheese samples, raising the possibility of contamination. However, the detection was limited to viral RNA; it did not establish the presence of live, infectious viruses, suggesting further research for food safety [88]. Furthermore, the UK Food Standards Agency’s 2024 risk assessment mentioned similar inadequate data on viral presence in colostrum-based cheese products, but was concerned about the potential for public health exposure via imported or specialty dairy products [89]. Quantitative risk models suggest that ingestion of small portions of contaminated cheese could result in infection. As H5N1 evolves in mammalian hosts and adapts to colder, non-respiratory environments such as milk and its derivatives, the dairy industry could unknowingly facilitate virus transmission not only on farms but across borders. Therefore, regulatory bodies, researchers, and public health communities should work together to address potential concerns at the consumer level. It is necessary to look beyond raw milk and raw-milk-based cheese, requiring robust testing of all cheese, yogurt, colostrum powders, and whey. Furthermore, global genomic surveillance of the virus in dairy products is necessary.

## 7. Occupational Exposure: A Significant Pathway for Virus Spillover to and from Humans

The emergence of HPAI H5N1 in U.S. dairy cattle has substantially changed the occupational risk landscape, pointing to farm workers as one of the important intermediaries in the zoonotic transmission of the virus, long thought to be primarily avian-bound. Dairy workers are particularly vulnerable due to close contact with infected animals, especially during high-risk tasks such as milking, feeding, and raw milk handling, where splashes, aerosols, and shared equipment significantly increase opportunities for mucosal contact with infectious materials. It is important to note that the use of Personal Protective Equipment (PPE) in dairy farms, such as face shields or respiratory masks, remains inconsistent and rarely standardized [90]. As of May 2025, at least 42 confirmed human H5N1 infections had been reported among dairy workers who had been in direct occupational contact with infected cattle across multiple U.S. states [58]. According to CDC and MMWR reports, as well as recent clinical observations, these infections have been predominantly mild, most often presenting with conjunctivitis, occasionally mild respiratory symptoms, and no fatalities. This pattern contrasts remarkably with earlier human H5N1 infections associated with poultry, where severe pneumonia and high mortality were typical, with a more than 50% case fatality rate [48,91]. Nevertheless, the recent poultry-associated cases further illustrate these differences. In U.S. poultry depopulation workers exposed to clade 2.3.4.4b, illness was similarly mild, consisting mainly of conjunctivitis, mirroring the cattle-associated risks to humans. Genomic analysis of viruses that infected bovines and humans in the U.S. dairy outbreak reveals mutations in hemagglutinin that enhance binding to human upper respiratory tract receptors (α2,6-linked sialic acids) and polymerase mutations that support replication at mammalian body temperatures [85]. However, no evidence of sustained human-to-human transmission has yet been reported.

Nonetheless, the persistence of a viable virus in environmental reservoirs such as bedding, equipment, and feces poses an indirect threat for workers and risks possible silent transmission, particularly in rural farm environments where shared labor, households, and cohabiting pets further complicate containment [92]. Moreover, the identification of H5N1 infections among domestic cats in the households of dairy workers triggers the possibility of occupational exposure beyond the dairy farms [58]. Similar poultry outbreaks have shown that indirect contact through contaminated surfaces or shared resources can lead to human infection, suggesting a parallel risk in cattle-associated outbreaks. Although human-to-human transmission has not been identified, investigations on ferret models in experimental studies demonstrated a transmission efficiency of up to 33% using virus isolates from an infected dairy worker [33]. It is speculated that while viruses are not yet fully adapted for efficient human spread, they may be undergoing genetic changes that enhance their capacity for human-to-human transmission. The silent spread of H5N1 in cattle and humans warns that infections without known exposure may signal viral adaptation, an alarming step toward sustained human transmission and pandemic risk [93]. The absence of structured One Health integration in the dairy industry may create a blind spot where a pathogen can silently adapt. Immediate interventions should include mandatory PPE; targeted occupational surveillance; enhanced virological monitoring of milk parlors, milk, and dairy barn surfaces; and real-time genomic tracking of strains circulating in dairy herds.

## 8. Recent Development of Immunization Strategies for HPAI H5N1 in Dairy Cattle

Since their introduction in 1945, influenza vaccines have played a vital role in curbing outbreaks from the 1918 Spanish flu to the 2009 A (H1N1) pandemic. Despite these advancements, vaccine efficacy remains inconsistent due to the virus’s rapid evolution via antigenic shift, drift, and reassortment [94]. However, the recent discovery of HPAI H5N1 clade 2.3.4.4b genotype B3.13 in U.S. dairy cattle highlighted a great concern in zoonotic disease ecology, demanding immediate attention to bovine-targeted immunization strategies. On the other hand, poultry vaccines continue to evolve, with improved antigenic matching and novel platforms. A recent study revealed that reverse genetics-derived inactivated vaccines, combined with advanced mucosal adjuvants, demonstrated an effective reduction in viral shedding and strong seroconversion, despite the challenges posed by antigenic drift in clade 2.3.4.4b viruses [95]. Meanwhile, lipid nanoparticle (LNP)-encapsulated mRNA vaccines encoding H5 hemagglutinin have shown promising results in SPF chickens [96]. Despite these advances, antigenic diversity and incomplete cross-protection remain substantial hurdles, prompting the development of multivalent constructs to extend vaccine coverage [97].

Bovines are recognized as novel mammalian hosts, capable of amplifying viral transmission to other species, including humans, which marks a critical reflection point in avian influenza control and prevention efforts. The unique immunobiology of ruminants, which is characterized by complex mucosal immune responses, the suppressive role of maternal antibodies in calves, and a lack of a defined correlation of protection, poses significant barriers to conventional vaccine design and efficacy assessments [57]. As a result, a recent study evaluated the immune response and milk antibody transfer in calves and lactating cows vaccinated with an inactivated clade 2.3.4.4b H5 avian influenza virus [98]. The results demonstrated a clear dose-dependent humoral response with peak hemagglutination inhibition (HI) titers reaching up to 9 log2 in calves receiving higher vaccine doses, associated with reduced viral shedding. In lactating cows, antibodies were effectively transferred into milk, with ELISA detecting immune responses as early as two weeks post-vaccination, sometimes even preceding serum seroconversion [98]. This highlights the potential of milk as a non-invasive matrix for surveillance. While mild local reactions were noted at higher doses, the vaccine showed good safety and immunogenicity, supporting future research into use in cattle against H5N1 transmission risks.

In another study, a bovine isolate of H5N1 (A/Bovine/Ohio/B24OSU-432/2024) was investigated and identified with dual receptor-binding affinity to both α2,3- and α2,6-linked sialic acids [99]. This provides preliminary yet significant insights into vaccination strategies against HPAI H5N1. In a parallel immunization trial, partial seroconversion and localized mucosal immune responses in mammary tissues were observed in response to an experimental H5 DNA vaccine vector in dairy cattle [100]. These results indicate that mucosal-targeted immunity is possible; however, the response was insufficient to fully prevent viral shedding or systemic disease. Another study compared responses to an avian-derived subunit vaccine with a bovine-adapted formulation, which revealed that the antigenic drift in bovine-specific H5N1 strains significantly reduced the effectiveness of traditional poultry vaccines [99]. Therefore, more research is necessary for vaccine development and optimization.

Although the cases are limited to dairy farms within the U.S., these findings highlight the urgency of developing cattle-specific vaccines, including mucosal-targeting vaccines, mRNA, or live-attenuated vaccines. Despite these exciting developments, significant hurdles remain. The H5N1 virus mutates rapidly, producing diverse strains that can evade immune protection. This antigenic drift requires ongoing surveillance and vaccine redesign to stay ahead of the virus. Practical challenges, such as delivering vaccines effectively to large cattle herds and maintaining cold-chain storage for mRNA vaccines, also require innovative solutions [101]. Moreover, regulatory and logistical coordination across sectors is essential to ensure vaccines reach the animals that need them promptly. Ultimately, vaccinating dairy cattle is no longer a random consideration but a frontline intervention essential to the One Health approach, to reduce viral amplification, environmental contamination, and cross-species transmission risk. These recent investigations with future possibilities shed light on the research scope and further combination efforts, which could reveal species-specific vaccines with targeted interventions.

## 9. Strengthening Pandemic Preparedness: Lessons from the HPAI H5N1 Outbreaks in Dairy Cattle and Future Directions

**Figure 6:**
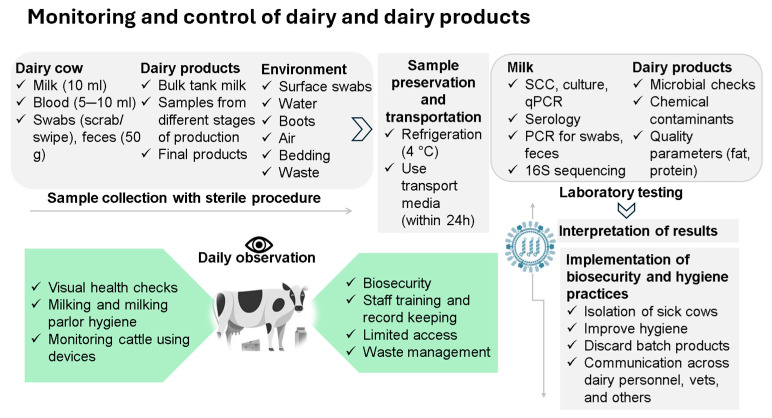
Schematic workflow for the monitoring and control of dairy cows and dairy products in the context of H5N1 surveillance. The diagram outlines comprehensive strategies beginning with sterile sample collection from cows, dairy products, and the environment, followed by preservation (refrigeration and transport media), laboratory testing (e.g., qPCR, serology, sequencing), and result interpretation. It emphasizes daily visual health monitoring, biosecurity enforcement, and staff training. The final step involves applying test results to enforce hygiene measures, isolate infected animals, discard contaminated products, and enhance cross-sector communication. Please note that Figure 6 has been constructed based on a literature review and recommendations are derived from authors’ professional experience.

The recent emergence of the HPAI H5N1 virus in U.S. dairy cattle presents a critical and unexpected development in the epidemiology of influenza viruses, exposing substantial gaps in our current understanding of host susceptibility, viral adaptation, and transmission ecology. Previously, IAVs were believed to infect birds and some mammals primarily, but recent reports from the United States indicate that dairy cows can also become infected and even shed the virus in their milk [102]. This unusual host jump raises many unanswered questions about the mechanisms facilitating cross-species transmission to bovines, intra-herd transmission dynamics, and the role of asymptomatic carriers in continuous outbreaks. In addition, viral shedding through raw milk introduces public health risks, particularly in regions where unpasteurized dairy consumption is common and pasteurization practices are inconsistently followed. Current testing methods, with the lack of validated diagnostic assays, may not fully detect the virus in cows since viral loads can vary between tissues, such as in milk, nasal swabs, and feces [103]. As the H5N1 panzootic virus expands across species barriers, innovative surveillance strategies are urgently needed to detect viral spread beyond traditional hosts (Figure 6). In this context, a very recent study demonstrated that amplicon sequencing of pasteurized retail milk can enable genomic surveillance of H5N1 in cattle, offering a novel, non-invasive tool for outbreak monitoring. However, its effectiveness is limited by low viral RNA levels and RNA degradation during pasteurization, warranting further validation in broader surveillance settings [104].

Furthermore, human exposure scenarios, especially among dairy farm workers, veterinarians, and milk processors, remain poorly investigated due to the absence of large-scale seroepidemiological studies [16]. While no severe human cases have yet been reported, the zoonotic potential of bovine-adapted H5N1 strains in humans remains unknown. On the other hand, the potential of cattle to act as “a mixing vessel” similar to swine raises concerns about viral evolution and pandemic potential. This can occur especially in mixed-species farming environments where co-infection with avian and mammalian influenza strains is plausible [83]. Alarmingly, there is still no approved vaccine for use in cattle against avian influenza, and it is unclear whether existing poultry or swine vaccines would be effective in cattle [57].

The environmental stability and shedding patterns of the virus in manure or contaminated fomites remain under-explored, which limits our understanding of farm-to-farm transmission risks [33]. Moreover, the role of farm wildlife, such as birds, rodents, or feral animals, in spreading the virus between farms has not been fully explored. These gaps in knowledge point to the urgent need for targeted research into how H5N1 behaves in cattle, how it might affect public health, and what steps are necessary to control future outbreaks. On a broader level, the socioeconomic implications, such as consumer fear, milk-trade restrictions, and disruptions in the dairy supply chain, could affect smallholder farmers and rural livelihoods, particularly in low and middle-income countries.

A serological analysis in naturally exposed dairy herds revealed robust and characteristic antibody responses to clade 2.3.4.4b HPAI H5N1 viruses, including subtype-specific neutralizing activity and NP-directed IgG [105]. These immune profiles indicate that cattle not only become infected but may also sustain antigenic exposure sufficient to trigger adaptive responses. These challenges exist in paradigms that classify bovines as epidemiologically insignificant in avian influenza transmission. Notably, the cattle-derived serological signatures differed from classical avian profiles, suggesting host-specific immunodynamics and warranting inclusion of ruminants in future One Health surveillance frameworks.

Nevertheless, special attention is needed to the spread of the virus to domestic pets, since several cat deaths have been reported from contaminated raw milk, cat foods, and dairy farm premises [78]. Although authorities have recommended limited access to unpasteurized raw milk or wildlife and dairy premises, there is still a question as to the virus’s potential for adaptation at the pet–human interface. As with the other challenges mentioned here, an urgent, coordinated response to protect animal welfare, public health, and economic stability should be implemented. Routine surveillance measures, such as bulk milk testing, have potential in detecting viral presence before clinical symptoms manifest, enabling timely interventions [33]. The CDC and USDA have issued upgraded protocols and guidance emphasizing the use of PPE, strict farm biosecurity, and enhanced surveillance protocols [90,106]. Surveillance efforts include weekly bulk milk testing, pre-movement screening, carcass and product sampling, and mandatory case reporting, which are essential for early detection and containment. Concurrently, biosecurity enhancements such as restricted farm access, disinfection of vehicles and equipment, minimizing wild bird exposure, disinfecting shared equipment, enforcing animal isolation protocols, and managing personnel movement between farms are crucial to limiting viral transmission. At the same time, mandatory PPE training, maintaining animal health records, and developing farm-specific outbreak preparedness should be implemented strictly to build resilience. In addition, ethical and economic challenges, particularly regarding the culling of valuable dairy herds and cross-state trade disruptions, highlight the need for humane, sustainable alternatives such as vaccination and genetic resistance research. Additionally, public awareness and education efforts are necessary to further strengthen outbreak management. Although the current outbreak events are confined to the United States, standardizing biosecurity protocols and fostering cross-sectoral collaboration are vital for a unified approach to disease management. Investing in research to develop cattle-specific vaccines and exploring genetic resistance to avian influenza in livestock can provide long-term solutions. Furthermore, the integration of artificial intelligence-driven analytics, including interconnected networks and behavior modeling of cows, may generate promising tools for real-time risk assessment of livestock herds to facilitate disease control. Together, these advances not only enhance early-warning capabilities at the animal–human interface but also provide actionable intelligence for vaccine deployment, biosecurity reinforcement, and One Health coordination.

## Data Availability

Not applicable.
